# Self-Assembly Behavior of Collagen and Its Composite Materials: Preparation, Characterizations, and Biomedical Engineering and Allied Applications

**DOI:** 10.3390/gels10100642

**Published:** 2024-10-08

**Authors:** Chengfei Yue, Changkun Ding, Minjie Xu, Min Hu, Ruquan Zhang

**Affiliations:** 1 State Key Laboratory of New Textile Materials and Advanced Processing Technologies, School of Textile Science and Engineering, Wuhan Textile University, Wuhan 430200, China; cfyue@wtu.edu.cn (C.Y.);; 2 Tianjin Key Laboratory of Advanced Fibers and Energy Storage, School of Materials Science and Engineering, Tiangong University, Tianjin 300387, China

**Keywords:** collagen, self-assembly, composite materials, preparation, applications

## Abstract

Collagen is the oldest and most abundant extracellular matrix protein and has many applications in biomedical, food, cosmetic, and other industries. Previous reviews have already introduced collagen’s sources, structures, and biosynthesis. The biological and mechanical properties of collagen-based composite materials, their modification and application forms, and their interactions with host tissues are pinpointed. It is worth noting that self-assembly behavior is the main characteristic of collagen molecules. However, there is currently relatively little review on collagen-based composite materials based on self-assembly. Herein, we briefly reviewed the biosynthesis, extraction, structure, and properties of collagen, systematically presented an overview of the various factors and corresponding characterization techniques that affect the collagen self-assembly process, and summarize and discuss the preparation methods and application progress of collagen-based composite materials in different fields. By combining the self-assembly behavior of collagen with preparation methods of collagen-based composite materials, collagen-based composite materials with various functional reactions can be selectively prepared, and these experiences and outcomes can provide inspiration and practical techniques for the future development directions and challenges of collagen-based composite biomaterials in related applications fields.

## 1. Introduction

The word “collagen” was coined in the 19th century from the Greek word “kola”, meaning “glue”. The other Greek word used as the suffix is “-gen”, meaning “production”, referring to the connective tissue component that produces gelatin when boiled and is thought to be the biological glue that holds cells in place [1]. However, the ancient Egyptians already used collagen to make paint and furniture adhesives more than 4000 years ago. In 1973, the silk book on prescriptions, *Prescriptions for 52 Diseases*, unearthed from the Mawangdui Tombs of the Han Dynasty, situated in Changsha, Hunan Province, China, was the earliest written record of human use of collagen. In the mid-19th century, based on the understanding of collagen’s unique amino acid composition characteristics, William Astbury first used X-ray diffraction (XRD) technology to study collagen molecules and believed that it was mainly composed of cis- and trans-peptides, which was the first time collagen was confirmed to have a regular structure. Ramachandra and Kartha then proposed a triple helix model based on the X-ray crystal diffraction image characteristics of collagen and its characteristic amino acid composition [2].In this triple helix model, three molecular chains are interwoven with the same central axis of the left-handed helix to form a right-handed triple superhelix [3,4]. With the continuous development of science and technology, scientists have made significant progress in the study of collagen structure, especially in determining collagen fibrils’ zonal periodic transverse stripe structure (D-band) [5]. At present, collagen is generally accepted by the international definition of ‘collagen is a structural protein of extracellular matrix, containing one or more domains with triple-helical structure [6]. Currently, 29 kinds of collagen have been discovered by comparing their homology with other collagen genes [1]. Type I collagen, the most abundant collagen in mammals, is the collagen discussed in this paper.

## 2. Structures and Properties of Collagen

### 2.1. Biosynthesis and Extraction of Collagen

With the development of bioengineering technology, the research on collagen biosynthesis has reached the gene level. Like other secreted proteins, the biosynthesis process of collagen is extremely complex, including the transcription and processing of collagen genes on cellular chromosomes, translation of genes into collagen molecules, secretion of collagen monomer and assembly into biofunctional microfibers in animal tissues, etc. [1,7,8,9,10]. In addition, the biosynthesized collagen microfibers must undergo various modification steps in the later stage and finally form different types of collagen with highly diverse biological functions in other tissues [11].

The earliest archeological evidence of human use of collagen was in Israel, and United Shoe Machinery successfully developed the commercial process for extracting collagen in 1962 [12]. The preparation process of collagen mainly includes material source selection, material pretreatment, and collagen extraction. After nearly 60 years of development, collagen preparation technologies are quite mature, breaking the limitations of the original complex process and numerous steps, and have developed in the direction of industrial production. In addition, the source of raw materials for collagen is gradually expanding, which not only diversifies collagen products but also helps reduce the preparation cost. The principle of collagen extraction is to separate collagen with different properties from different tissues by changing the external environment, such as temperature, salt concentration, and pH [13]. Collagen extraction methods are mainly divided into chemical methods (alkali, salt, and acid extraction methods) and enzyme methods [14,15,16].

Chemical methods extract soluble collagen that has not matured or failed to be covalently cross-linked with tissues. Alkaline extraction methods are based primarily on the saponification of fats bound to collagen with an alkaline solution to remove terminal peptides and dissolve collagen without helices [13]. However, the alkaline process is complicated and quickly leads to the hydrolysis and racemization of peptide bonds, seriously affecting the product collagen’s relative molecular weight and biological application [13]. Salt extraction methods are achieved by the salt dissociation of ion bonds between peptide chains to cause the salt swelling of collagen fibers, thereby completing the dissolution of collagen fibers [17]. In salt extraction methods, due to the difference in the lyotropic series (Hofmeister series) caused by different salts, the process of salt extraction is precarious, which also affects the physical/chemical properties of collagen to varying degrees [18,19]. Acid extraction methods are the most widely used chemical extraction method of collagen, which mainly relies on the low ion concentration under acidic conditions to destroy the ionic bonds between collagen molecules, resulting in the swelling and dissolution of collagen fibers, thus achieving the purpose of collagen extraction [20]. Under the strict control of hydrolysis temperature, time, acid concentration, and other extraction conditions, the acid extraction methods of collagen can maintain a complete three-strand helix structure, which is suitable for applying biomedical materials [11]. Enzymatic methods are commonly used to extract insoluble collagen covalently cross-linked in tissues. Since most covalent bonds are located in the N-terminal and C-terminal peptide parts of insoluble collagen, proteases other than specific collagenases can degrade collagen under certain conditions [21]. Either papain under neutral conditions or pepsin under acidic conditions can remove the non-helical end of collagen, making it soluble. In addition, pepsin can also cause the development of α_1_ and α_2_ chains in collagen molecules, reducing the antigenicity of collagen.

In the process of collagen extraction, there is a specific upper limit of the collagen extraction rate due to the existence of soluble and insoluble collagen. In actual production, researchers often combine multiple extraction methods to improve the collagen extraction rate [16]. It is worth noting that the hot water extraction methods mentioned in some literature cannot obtain natural collagen, but denatured collagen or collagen hydrolysate gelatin can. Moreover, in addition to the traditional extraction methods discussed above, in recent years, researchers have used recombinant DNA technology (i.e., genetic engineering technology) to shear the genes involved in collagen synthesis into different host cells (such as yeast, cattle, or sheep cells) to produce collagen [22]. Luo et al. cut the human collagen gene fragment into *E.coli* and significantly increased the secretion of human-like collagen by optimizing the composite medium [23].

### 2.2. Structure of Collagen

The spatial structure of collagen is the basis for expressing biological function and showing the relationship between structure and function [24]. The primary structure of collagen refers to the linear sequence of interconnecting peptide bonds, that is, the number, type, connection mode, and arrangement order of amino acids that make up the peptide chain, which also determines the high-grade structure of collagen. The α chain of collagen comprises 20 amino acids arranged repeatedly in the (Gly-X-Y)_n_ principle, where X and Y represent any amino acid except glycine. In addition, the amino acid residues of X and Y can also determine the triple helix structure of collagen. The secondary structure of collagen usually refers to the local regular folding of the collagen α chain, that is, the formation of a locally ordered spatial structure of adjacent amino acids in the peptide chain [25]. Unlike the α-helix and β-fold common in most proteins, the secondary structure of collagen forms a left-handed α-helix due to electrostatic repulsion between amino acids at the X and Y positions [24]. In addition, the amino acid residues on the side chain form hydrogen bonds within the α-helix to maintain the α-helix structure of the polypeptide chain [25]. On this basis, the three left-handed α-helical polypeptide chains bend, fold, and intertwine to form a right-handed superhelical structure called the tertiary structure of collagen, also known as procollagen, depending on the secondary bonding between peptide chains (including ionic bonds, hydrogen bonds, van der Waals forces, and hydrophobic bonds, etc.) [24]. As the basic structural unit of collagen, the rotation direction of its three-strand helical structure is opposite to the rotation direction of the α chain, so it is not easy to unspiral, which makes collagen have extremely high strength in biological tissues. The quaternary structure of collagen refers to the formation of collagen microfibrils, which are formed through covalent bonds between the head and tail, to form a specific distance of longitudinal symmetry staggered by one-fourth of a molecule length and then further arranged in parallel bundles to form stable collagen fibrils. The regular distribution of amino acids in the α chain of collagen leads to the periodic alternation of polar and non-polar regions, resulting in a 64–67 nm D-band in collagen fibrils, which is also a sign of the high degree of self-assembly of natural collagen [26].

### 2.3. Properties of Collagen

Collagen is a kind of biological macromolecule, and the hydrophilic groups on its peptide chain can hydrate with water in an aqueous solution, wrap a hydration layer around the procollagen, separate the procollagen from each other, and form a stable liquid sol, showing colloidal properties [3,27]. In addition, collagen, which takes amino acids as its basic structural unit, is also a polyvalent ion. Collagen molecules with the same charge repel each other and can form stable double electric layers with ions with opposite charges around them to prevent the mutual aggregation of molecules, which also enhances the stability of the collagen solution [20].

Collagen is not only a polyvalent ion but also an amphoteric polyelectrolyte. The collagen solution has an isoelectric point (pI), which makes the net charge of the collagen surface zero and exhibits special physical and chemical properties. The pI of collagen will vary depending on the source or extraction method, and common testing methods include Zeta potential, fluorescence spectrophotometry, and isoelectric focusing disk electrophoresis [28]. The net charge of collagen at pI is zero, and the molecule is electrically neutral and tends to curl, showing the property of minimum viscosity of the solution. When the pH of the collagen solution deviates from pI, the net charge of collagen is not zero, and the molecular chain has a positive or negative charge. Electrostatic repulsion tends to stretch the collagen molecular chain, and the side chain groups of the molecular chain are more easily exposed, which allows the molecular chains to increase the viscosity of the collagen solution by entangling and binding with each other. Another characteristic of collagen at pI is the minimum swelling. There are two main theories of the pH swelling mechanism of collagen: equilibrium osmotic swelling and electrostatic repulsion. The equilibrium osmotic swelling theory holds that collagen has colloidal properties in an acid-based solution and is a semi-permeable membrane through which the electrolyte outside the membrane can freely combine with the amino acid residues of collagen. When osmosis reaches dynamic equilibrium, the electrolyte concentration inside collagen is greater than that outside collagen, resulting in water infiltration into collagen and swelling. According to the electrostatic repulsion theory, the side groups on the collagen peptide chain will repel each other due to the same charge when the pH of the solution deviates from pI, which increases the distance between the peptide chains and causes swelling [29].

The thermal properties of collagen are essential factors affecting its structure and properties, including thermal shrinkage temperature and thermal denaturation temperature. The temperature at which collagen shrinks axially to about 5% of its original value when heated is called the heat contraction temperature. On the other hand, the collagen triple helix unfolds when heated and gradually forms a single chain, and the temperature at which the triple helix structure dissolves to about 50% is the thermal denaturation temperature of collagen [30]. The main factors affecting the thermal properties of collagen are the synergistic effects of various intramolecular and intermolecular interactions, such as hydrogen bonds, disulfide bonds, van der Waals forces, ionic action, hydrophobic action, and hydration [1,26,31,32,33,34]. In addition, the source of collagen (including animal species, age, living environment, etc.), amino acid composition and sequence, electronic effects, and supramolecular assembly are also important factors affecting the thermal properties of collagen [10]. Sinthusamran et al. extracted collagen from the skin and swim bladder of seabass by acid methods and found that the thermal stability of fish swim bladder collagen with high amino acid content (proline and hydroxyproline) was higher than that of skin collagen by comparison [35]. Liu et al. also reported that the thermal stability of collagen in bighead carp tissues (swim bladder and bone) was slightly higher than that of in vitro tissues (fins, scales, and skin) [36].

Collagen is responsible for the elasticity of biological tissues in animals, and its mechanical properties are mainly related to its chemical composition, intramolecular and intermolecular cross-linking, and helical structure [37]. The length and diameter of collagen fibrils, spatial distribution, collagen type, non-collagen molecule content, and degree of cross-linking also determine the function and mechanical properties of skin, tendon, cornea, blood vessels, cartilage, bone, and other tissues [38]. Researchers believe that non-collagen components such as elastin interact with collagen fibers through their unique viscoelasticity, allowing tissues to withstand compression and tensile forces [39]. In addition, the deformation mechanism of collagen is similar to that of crystalline polymers that generate plastic flow, which can be divided into toe zone, heel zone, linear zone, and fracture zone [40,41]. For collagen-based composite materials, the multistage structure of collagen is extensively deformed during the loading process of large strain, and the fibril splits into a single microfibril. The stretching of the three-strand helical structure and the side-by-side sliding of microfibrils are possible mechanisms for converting mechanical energy into fiber deformation during the stretching process [1,24,42].

As an important animal protein, collagen’s biological properties mainly include low immunogenicity, cell–matrix interaction, coagulation effect, fiber reformability, and biodegradability. On the other hand, as the adhesive substance of connective tissues (ligaments, tendons) in animals, collagen provides stable support for related tissues in the body through these physiological properties so that they can give full play to their corresponding physiological functions.

## 3. Self-Assembly Behavior of Collagen

Molecular self-assembly is common in biology and can form various complex biological structures. In fact, the synthesis of collagen fibril in vivo is also a self-assembly process determined by the intrinsic properties of collagen molecules and cell-mediated biological regulation. This highly organized collagen fiber structure from the nanoscale to the macroscale provides excellent thermal stability, mechanical properties, and various biological functions for connective tissue. On the other hand, great attention should also be paid to the interactions between biomaterials and cells when designing collagen-based biomaterials. Many previous works have suggested that the self-assembly kinetics and architectures of collagen materials, which closely depend on the in vitro incubation conditions, determine the distinctive cell fates [26]. Although researchers have made great strides in the in vivo regulation of collagen fiber formation and have acquired a broad theoretical basis, the cellular mechanism of fibrous tissue in vivo has remained elusive [43]. This complexity can be attributed to the dynamic nature of in vivo self-assembly during stages such as tissue development and tissue aging. Therefore, given the heterogeneous and dynamic nature of collagen fibril formation in vivo, systematically characterizing these incubation conditions allows for a more rational design and control of the structure and morphological features of collagen-based biomaterials on a molecular scale, which could help to obtain varying stable collagen fibrillogenesis products for tissue engineering applications [26].

### 3.1. Self-Assembly Mechanism of Collagen

In biological organisms, collagen can spontaneously assemble into ordered collagen microfibers and fiber bundles and then form macronetwork structures, which are the structural framework of many biological tissues and can give tissues excellent biomechanical properties and diverse organizational structures [26]. Researchers have recently found that collagen molecules can self-assemble into ordered aggregates or supramolecular structures in vitro by properly regulating the external environment and eventually forming fibers [44,45,46]. The formation of this structure is based on the molecular recognition between collagen molecules or between collagen molecular fragments through the synergistic effect of many non-covalent bond weak interaction forces, including hydrogen bonds, hydrophobic forces, electrostatic forces, van der Waals forces, π–π stacking forces, etc. [34,47]. This process is also closely related to collagen’s inherent three-strand helical structure, unique sticky terminal, and molecular chirality [26,48].

At present, a typical school of thought based on the theory of “nucleation-growth” has been established to study the mechanism of the collagen self-assembly process in vitro [26,49]. As early as the 1950s and 1960s, Wood et al. explored the dynamic process of collagen self-assembly by using the turbidity method and proposed the theory of “nucleation-growth”, which divided the self-assembly process of collagen molecules into two stages: initiation and growth [50,51,52]. In the initial stage, collagen molecules interact with each other to form collagen microfibers. In the growth stage, these collagen microfibers rapidly form collagen fibril through lateral and axial polymerization, and the whole process conforms to the S-shaped curve. Goh et al. used light scattering technology and atomic force microscopy (AFM) to study the self-assembly process of collagen from molecules to fibrils and proposed the stepwise assembly theory, which was consistent with the “nucleation-growth” theory proposed by Wood et al. [53]. It is precisely due to these structural properties of collagen molecules and the driving force provided by the coordination of molecular forces that collagen molecules can accurately assemble to form a highly ordered supramolecular structure. Collagen self-assembly to form highly ordered biological functional materials is conducive to cell adhesion, proliferation, diffusion, and migration and can be used as a vital tissue substitute material [26,54,55].

### 3.2. Factors Affecting Collagen Self-Assembly

It has been widely demonstrated that collagen molecules can self-assemble into fibrils with typical banded structures at a suitable temperature and in a neutral salt solution. The main factors affecting the in vitro self-assembly of collagen molecules are concentration, temperature, pH, substrate, ionic strength, etc. These factors often change the formation of the “core” during nucleation by influencing the interactions between molecules (hydrophobic interactions, hydrogen bonds, electrostatic interactions), thus changing the formation of collagen fibrils. Therefore, it is of great significance to understand the influencing factors of collagen self-assembly for designing and preparing collagen-based biomaterials.

#### 3.2.1. Collagen Concentration

Concentration is one of the main factors affecting collagen self-assembly behavior in vitro. According to DLVO theory, there are both repulsive potential energy and gravitational potential energy between collagen molecules, which makes the stability of collagen solution depend on the relative magnitude of the repulsive potential energy or gravitational potential energy. In the solution with a low collagen concentration, the number of collagen molecules per unit volume is small, and the repulsive potential energy between collagen molecules is relatively more significant than the gravitational potential energy, which will hinder the “collision” between collagen molecules caused by Brownian movement and keep the collagen solution in a relatively stable state. With the increase in the collagen concentration, the number of collagen molecules per unit volume increases, resulting in the gravitational potential energy between molecules being relatively more remarkable than the repulsive potential energy, resulting in the collagenous molecules moving closer to each other and undergoing self-assembly. For the research on the mechanism and influencing factors of collagen self-assembly, the concentration of collagen dilute solution used is generally lower than the critical microfiber concentration, and the self-assembly behavior is induced by changing the external conditions. Yan et al. [48] explored the influence of the concentration on the in vitro self-assembly dynamics of cod collagen by using the turbidimetric method. The results show that the self-assembly process of collagen is consistent with the “nucleation-growth” theory, which refers to the formation and growth of the “core”. Significant self-assembly occurred when the collagen concentration was greater than 0.6 mg/mL, and increasing the concentration accelerated core formation and nucleus growth. In contrast, no considerable self-assembly was observed below this concentration. Fang et al. [56] found that no fibrous structure could be observed under AFM when collagen concentration was lower than 0.5 mg/mL, while fibrils with D-band could be observed when collagen concentration was higher.

#### 3.2.2. Temperature

Collagen is heated above its denaturation temperature as a heat-sensitive protein, causing its triple helix structure to curl freely. However, collagen molecules can self-assemble under the right conditions when the temperature is lower than the denaturation temperature. Huelin et al. [57] showed that collagen could self-assemble at 25–37 °C and near-neutral pH. In addition, an increase in temperature below the denaturation temperature can accelerate the self-assembly process, forming more tightly structured collagen fibrils [58]. Amino acids on collagen and surrounding water molecules will combine with hydrogen bonds to form hydration layers. When the temperature increases, the kinetic energy of water molecules increases, and the displacement of hydration layers will promote collagen self-assembly behavior [45,59]. This means that the self-assembly process of collagen is endothermic [60]. Based on this, many studies on the control of the collagen self-assembly process and the morphologies of self-assembled collagen fibrils by adjusting culture temperature have provided a theoretical basis for collagen self-assembly in vitro [61,62].

#### 3.2.3. pH

The polypeptide chains that make up collagen molecules have different charges, and pH will affect the state of charge of different peptide chains, thus affecting the electrostatic interaction and self-assembly behavior between collagen molecules [48]. Therefore, by adding different charged substances, the state of charge of collagen molecules can be changed, and the competition among collagen molecules, ions, water molecules, and substrate can be regulated, thus affecting the self-assembly structure of collagen.

**Figure 1:**
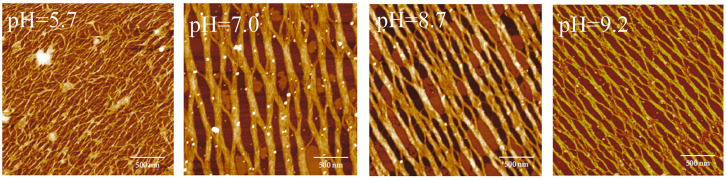
Typical AFM height images of collagen self-assembly at different pH values [63].

Song et al. [63] used AFM to observe the self-assembly behavior of the swine trotter tendon under different conditions. The width of collagen fibrils was first increased significantly and then decreased with the increase in pH (Figure 1). Yadavalli et al. [64] studied the in vitro self-assembly behavior of collagen molecules under different pH conditions and observed the morphologies of self-assembled collagen fibrils through AFM. The results showed that no significant collagen self-assembly occurred at pH 3.0 for several days. The self-assembly of collagen occurred at pH 5.0, 7.0, and 9.0, and the rate of collagen molecules forming fibrils at pH 5.0 was much slower than that at pH 7.0 and 9.0. Dehsorkhi et al. [65] also conducted a systematic study on the effect of pH value on the self-assembly of collagen-simulated peptides and found that, when the pH gradually increased, the self-assembly morphology changed from globular particles and flat strips to winding fiber strips and flat wide strips.

#### 3.2.4. Substrates

**Figure 2:**
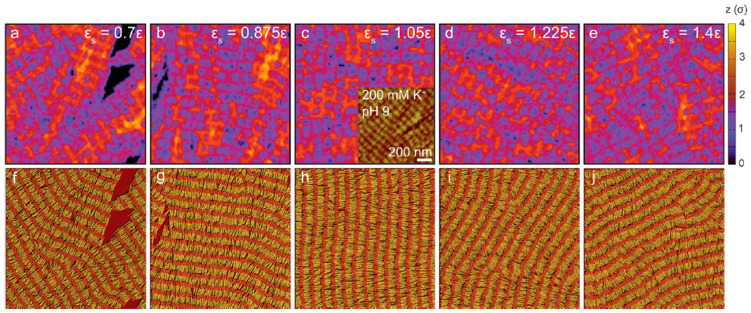
Molecular dynamics study of the effect of the strength of collagen–substrates interaction on the morphology of the deposited collagen molecules during postdeposition heat treatment [67].

Over the past two decades, collagen adsorption and assembly studies at the interface have increased significantly as the development of AFM and related technologies have revolutionized our ability to observe adsorbed proteins at the (supermolecular) scale and in different media. Understanding and controlling the adsorption and self-assembly of collagen at the interface is of great significance to the study of the self-assembly behavior of collagen [66]. The substrate materials commonly used in the collagen self-assembly process include glass sheets, mica sheets, silica, polystyrene, etc. Due to the anisotropy and amphiphilicity of collagen molecules, the lattice arrangement, surface chemical properties, charge characteristics, and hydrophobicity of substrate materials will greatly impact the self-assembly of collagen [26]. The morphologies of self-assembled collagen fibrils are usually studied on certain base materials. When the dilute collagen solution used is self-assembled under certain conditions, the morphologies of the self-assembled collagen fibrils can be observed by AFM, and the self-assembly mechanism can be inferred. Narayanan et al. [67] studied the self-assembly behavior of collagen on a flat substrate and established a microscopic model to show that the morphologies of self-assembled collagen fibrils on a flat substrate are determined by the competition mechanism between collagen–collagen and collagen–substrate interactions. Figure 2 shows both the height variations for structures (a–e) and their corresponding bead structures with the two bead types identified by different colors (f–j). We can note that the height variations (a–f) correspond closely to the regions where the strongly interacting type 2 beads are together [67]. In systems where the collagen–substrate interactions are dominant, the movement of collagen molecules on the substrate is strongly hindered, which will restrict the lateral fusion and lateral connection of collagen microfibrils and hinder self-assembly into network structures. On the contrary, collagen molecules tend to self-assemble into network structures when collagen–collagen interactions dominate. Fang et al. [56] discussed the self-assembly behavior of collagen on the substrate materials of golden mica, muscovite, and molybdenum disulfide. It was found that due to the different lattice structures and symmetry of the substrate materials, the collagen fibrils on the substrate materials of gold mica, muscovite, and molybdenum disulphide show different self-assembled structures, which are triangle, oblique parallel lines, and network structure, respectively.

In recent years, the self-assembly behavior of collagen at special interfaces has become a hot topic in the field of collagen self-assembly behavior. Dupontgillain et al. [66] compared the self-assembly behavior of collagen on polyvinyl oxide (PEO), polystyrene (PS), polymethyl methacrylate (PMMA), PS/PMMA, and other substrates. It was found that collagen molecules formed sparse network structures on PEO, monolayer smooth fiber structures on PMMA, a large number of fiber structures on PS, and heterogeneous structures on PS/PMMA substrates. At the same time, it was found that the concentration of collagen fibrils on PS was higher than that on PMMA, which was mainly related to the hydrophobic effect of the substrate.

In addition, the in vitro self-assembly behavior of collagen is difficult to precisely control to produce a self-assembled structure with a defined size, shape, and fixed position on the substrate. Some studies have found that the position, size, and shape of collagen self-assembly can be determined by applying applied electric and magnetic fields, etc., which also opens up ideas for the preparation of biomimetic materials based on collagen self-assembly behavior to solve medical and biological problems [26,68].

### 3.3. Characterization Methods of Collagen Self-Assembly

It is well known that collagen molecules can spontaneously undergo obvious changes in solution transmittance and morphology during self-assembly. Based on this, several corresponding transmission, imaging, and mechanical methods have been used to study collagen fibril formation. Because the process and structure of molecular self-assembly are macroscopic and special, the characterization methods of molecular self-assembly are varied. The characterization methods of collagen self-assembly can be roughly divided into electrochemical characterization methods, microscopic characterization methods, and spectroscopy characterization methods.

The most common electrochemical characterization method for collagen self-assembly is zeta potential analysis. The charge of the collagen molecules itself is a crucial factor in determining the self-assembly process of collagen [69]. Zeta potential analysis can be used to study the influence of electrostatic interaction on the self-assembly process of collagen [47,70]. In addition, Friess et al. [71] also studied the in vitro self-assembly behavior of collagen by using the oscillating current test method.

Microscopic characterization technology is the most intuitive characterization method to display the microscopic morphology of collagen self-assembly. With high sensitivity and accuracy, it can obtain the electronic image, arrangement orientation, and structural morphology of the materials formed by self-assembly, which is suitable for characterizing the morphologies and structures of collagen self-assembly. AFM was widely used to observe the aggregation morphologies of collagen self-assembly [72,73,74]. Stamov et al. [75] successfully monitored the formation process of collagen fibrils in real-time in an in situ system on atomic-level planar mica substrate via AFM fast scanning imaging technology and studied its self-assembly dynamic process. Nudelman et al. [76] observed the mineralization process of collagen fibers by hydroxyapatite (HAP) using transmission electron microscopy (TEM). De Mesquita et al. [77] used scanning electron microscopy (SEM) to observe the surface and sectional structures of the self-assembled collagen–cellulose films with nanowhisker layers.

Spectroscopy characterization techniques are widely used to characterize the chemical structure of self-assembled collagen fibrils because they are highly sensitive and do not damage collagen structure [78]. There are many carbonyl, carboxyl, and amide groups with double-bond structures on collagen polypeptide chains. Fourier infrared spectroscopy (FT-IR), UV–visible absorption spectroscopy (UV-vis), and circular dichroism spectroscopy (CD) can be used to analyze and identify the self-assembly process of collagen according to the characteristic peaks of the corresponding groups [79,80,81].

## 4. Collagen-Based Composite Materials

**Figure 3:**
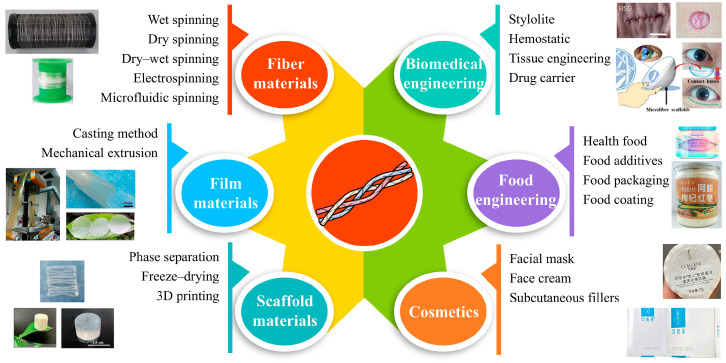
Main preparation methods and application fields of collagen-based composite materials.

Over the past decades, great achievements have been made in researching and developing biological materials, which have become essential for safeguarding human health. With the continuous economic development of society, the increase in the aging population, and the improvement of the quality of life, the chronic diseases plaguing human beings have become increasingly prominent, which makes people’s demand for biomedical materials increasingly large, especially for joints, artificial teeth, cardiovascular health, and other biomedical materials. However, the development of biomaterials is slow due to some of their shortcomings (the corrosiveness and allergenicity of materials, the need for secondary surgery, etc.). Collagen has become one of the most important biological materials due to its excellent biological properties and abundant sources. At the same time, as shown in Figure 3, collagen is easy to process, form, and can be made into many different forms of materials (such as membrane, fiber, sponge, gel, etc.), which has a wide range of applications in food packaging, daily chemical industry, and biomedical materials.

### 4.1. Preparation of Collagen-Based Composite Materials

#### 4.1.1. Collagen-Based Fiber Materials

**Figure 4:**
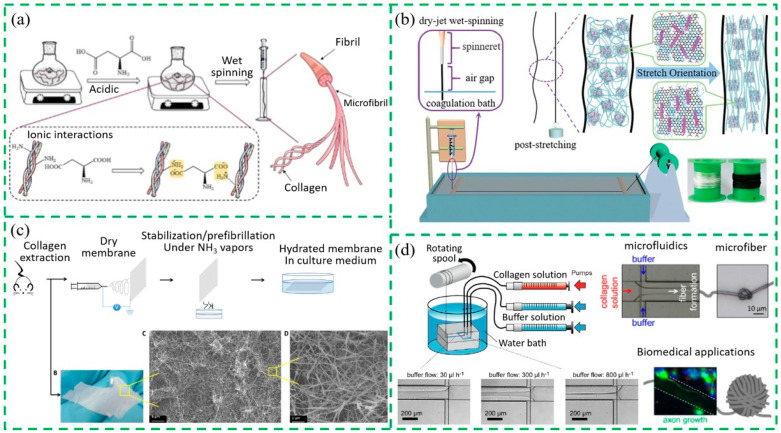
(**a**) Preparation of collagen/Asp composite fibers by wet spinning [72]; (**b**) preparation of collagen/GO-MWNT composite fibers by dry-jet wet spinning [90]; (**c**) scheme of collagen electrostatic spinning and membrane processing [91]; (**d**) microfluidics-produced collagen fibers [98].

The main manufacturing methods of collagen-based fiber materials include wet spinning, dry–wet spinning, electrostatic spinning, and microfluid spinning [82,83,84,85,86]. Myere et al. [87] used wet spinning and melt spinning to prepare collagen fibers and made a detailed comparison of the structure and physical and biochemical properties of the collagen fibers. The results show that the process of wet spinning is much more difficult than that of melt spinning. This is because in the process of wet spinning, not only the solid content of collagen spinning stock solution is low, but also the primary fiber needs to be dehydrated and precipitated for a long time in the coagulation bath, and the fiber surface is rough due to the effect of coagulation and double diffusion. On the other hand, the strength of collagen fibers prepared by wet spinning is higher than that of melt spinning, while the processing process of modified thermoplastic collagen is more complicated than that of the wet spinning of the collagen solution. Yue et al. [72] investigated the self-assembly behavior of collagen molecules in the presence of aspartic acid (Asp) and prepared collagen/Asp composite fibers using wet spinning. Their results showed strong molecular interactions between the negatively charged Asp and the positively charged collagen molecules in acidic conditions (Figure 4a). Green et al. [88] prepared multi-scale collagen/nanocarbon composite fibers by the gel spinning method and explored the effects of single-walled carbon nanotubes (SWNT) and cellulose nanocrystals (CNC) on the arrangement of collagen molecules and fibrils. For SWNT and CNC nanofillers with similar properties and inherent rigidity, the excellent dispersion quality facilitates the self-assembly behavior of the collagen fibril during fiber processing and also plays a vital role in the morphologies and mechanical properties of the resulting fibers. Ding et al. [89] prepared collagen/multi-walled carbon nanotubes (MWNT) composite fibers using dry–wet spinning technology and found that the diameter and length–diameter ratio of MWNT affected the self-assembly structures and microfibril arrangements in the process of collagen fiber forming, and the strength of composite fibers was significantly higher than that of pure collagen fibers, reaching 2.5 cN/dtex. Yue et al. [90] reported that graphene oxide (GO)-MWNT nanomaterials were constructed by using GO-dispersed MWNT, and the strength and toughness of collagen fibers were significantly improved by introducing GO-MWNT nanomaterials into the collagen matrix during dry-jet wet spinning that exhibited the advantage of positive stretching to the extruded fiber before coagulation (Figure 4b).

With the development of nanotechnology, electrospinning, as a unique nanofiber processing technology, plays a vital role in biomedical materials, filtration and protection, energy, medical beauty, and so on [84]. Dems et al. [91] used electrospinning, an extremely promising method for preparing fibrillar membranes, to mimic the extracellular matrix of native tissues. Dry electrospun collagen can be stabilized by prefibrillation in a vapor state to avoid collapse upon hydration, and this provides very easy-to-handle biomaterials of pure collagen that are ready to be used for biomedical applications, including implantations, with a preserved hierarchical structure and biological activity (Figure 4c). Zimba et al. [92] functionalized GO nanosheets through carboxylation modification to improve the combination of GO and collagen molecules. Collagen/carboxylated GO nanocomposite films were successfully prepared by electrospinning. Compared with pure collagen films, the addition of carboxylated GO deepens the appearance color of collagen and improves the mechanical properties of collagen films.

Since traditional spinning techniques have poor control over the fiber-forming process, the preparation of biological fibers can be carried out in a well-designed, simple, and economical way by combining microfluidic techniques with these traditional spinning techniques [93]. Microfluidic spinning technology is a new material preparation technology platform that has emerged recently. Its principle is similar to that of the spider spinning process. Unique microchannel design can precisely control the morphology, size, anisotropy, microstructure, and biological activity of materials [94]. Based on the hydrodynamic focusing effect, macromolecules in spinning fluid can fully stretch, align, and orient in strong tensile flow fields in microchannels [95]. Microfluidic spinning technology, which does not require a high temperature and high voltage and has a low energy consumption and mild reaction conditions, has attracted great interest in academia and industry due to its high-performance artificial fibers that can be designed and prepared through its structure. [96]. Koster et al. [97] conducted in situ studies on the self-assembly and dynamic evolution of collagen molecules in microfluidic devices and obtained collagen fibers with self-assembly structures through hydromechanical focusing to form oriented fibers. The collagen fibers prepared by this technique can enhance the mechanical properties of bone and tissue and affect the movement and morphology of cells. Haynl et al. [98] proposed a method based on microfluidic technology that can continuously produce adjustable collagen fibers with a tensile strength and Young’s modulus surpassing traditional wet-spun fibers and natural tendons, with smaller diameters (Figure 4d). FT-IR results showed that the orientation of collagen fibrils is the main reason for its excellent mechanical stability.

#### 4.1.2. Collagen-Based Film Materials

**Figure 5:**
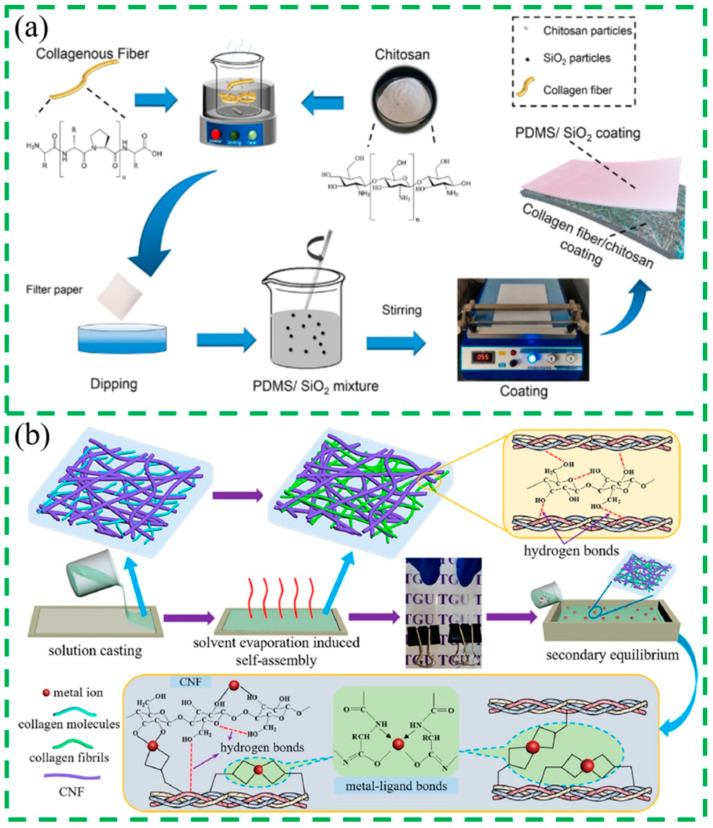
(**a**) The preparation process of collagenous fiber/chitosan-PDMS paper-based material [102]; (**b**) collagen composite films via the synergy of hydrogen and metal–ligand bonds [103].

With the continuous development of science and technology, film technologies in biomaterials have become an important branch of film science. Collagen-based film materials are widely used in food packaging, medical materials, and other fields due to their unique physical and chemical properties and excellent biological properties [99,100,101]. Collagen has good film-forming properties and can be prepared by mechanical extrusion and solution casting. During mechanical extrusion, the molecular chains inside the membrane are completely unfolded by the molding device by applying mechanical force to the membrane, facilitating the interaction between collagen molecular chains. Therefore, the mechanical strength of the membrane prepared by the mechanical extrusion method is higher. The casting film-forming method is formed by the flow of the casting liquid itself, which makes most of the molecular chains inside the film in a curly state, and the interaction between the molecular chains is less, ultimately affecting the film’s mechanical properties. Compared with the casting film-forming method, although the collagen film prepared by the mechanical extrusion method has higher uniformity, the complexity of the mechanical extrusion process and the lengthy technological process make the casting film-forming method become the main forming method of collagen films. Jing et al. [102] prepared a type of non-fluorine hydrophobic and oil-resistant material using collagen fiber, chitosan, and polydimethylsiloxane (PDMS) as raw materials (Figure 5a). Notably, 10 g/m^2^ of cross-linking product and 6 g/m^2^ of PDMS had a low pore size and a smooth and uniform surface, which made the composites exhibit excellent hydrophobic and oil-resistant properties (water contact angles of 141°), water and oil resistance (kit rating value of 12/12), and mechanical properties. Yue et al. [103] prepared collagen/cellulose nanofiber (CNF) composite films using solution casting-solvent evaporation method induced the self-assembly of collagen molecules in the CNF supramolecular network to form a high-density physically cross-linked supramolecular double network and then moved to inorganic saline solutions to induce the structure rearrangement and cross-linking of the composite films on the nanoscale through soaking (Figure 5b). Yang et al. [104], for the first time, prepared collagen films with different orientations using reverse rotary extrusion technology and demonstrated the positive role of the obtained collagen films in the culture of bone marrow mesenchymal stem cells in vitro and in the regeneration of the tendon in vivo by using rat Achilles tendon defect models. Lu et al. [105] used polyvinyl alcohol and collagen as raw materials, degraded collagen into gelatin through thermal/mechanical chemical action in the extruder, successfully prepared polyvinyl alcohol/gelatin composite films, and studied the influence of extrusion temperature on collagen degradation and the thermal stability and mechanical properties of composite films. The results showed that the controllable degradation of collagen in the extruder could be achieved by adjusting the extrusion temperature, which promoted the generation of low molecular weight gelatin, the esterification reaction between polyvinyl alcohol and gelatin, and the formation of hydrogen bonds so that the gelatin and polyvinyl alcohol had good compatibility. Andonegi et al. [106] prepared collagen/chitosan composite films by the casting film-forming method and evaluated the influence of chitosan with different molecular weights on the physical and chemical properties, thermal stability, and mechanical properties of collagen films. By controlling the pH value of the collagen solution, Xu et al. [29] prepared collagen films with different swelling degrees by using the casting film-forming method and explored the influence of pH value on the structures and properties of collagen films. The collagen solution will fully swell at the suitable pH value, and the collagen fibrils formed will form collagen films with a layered network structure through extensive swelling and winding.

#### 4.1.3. Collagen-Based Scaffold Materials

**Figure 6:**
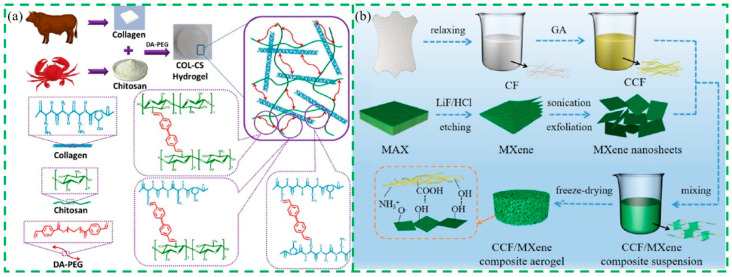
(**a**) Construction mechanism of collagen/chitosan hydrogels [110]; (**b**) fabrication procedures of collagen fiber, MXene, and collagen fiber/MXene composite aerogels [112].

A common scaffold fabrication technique is to form a hydrogel and then freeze-dry it [107]. Hydrogel is a kind of special semi-solid three-dimensional network polymer material that can swell in water and retain a significant amount of water without dissolving. Hydrogels are closer to living tissues than any other synthetic biological material, similar in nature to extracellular matrix parts, and can significantly improve the biological properties of materials by reducing friction and mechanical effects on surrounding tissues after absorbing water. The preparation methods of collagen hydrogels are mainly divided into physical cross-linking and chemical cross-linking. Physical cross-linking refers to collagen hydrogels formed by hydrogen bonds, coulomb forces, coordination bonds, and physical entanglement. Chemical cross-linking refers to the formation of a firm collagen hydrogel network by adding some functional groups on the collagen molecular chain to cross-linking agents such as glutaraldehyde (GA), genipine, and carbonized diimide, which can trigger chemical reactions such as addition and condensation between collagen itself or other polymers. Moxon et al. [108] prepared alginate/collagen hydrogel by fusing collagen fibrils into alginate saline gels by physical mixing and controlled gelation under physiological conditions and demonstrated that by adjusting the physicochemical properties of the alginate/collagen mixture, different qualitative microenvironments could be created to support the growth and development of human neuronal cells. Bai et al. [109] introduced the natural material tannic acid into the polyvinyl alcohol/collagen dual network to prepare a composite hydrogel with good biological activity and mechanical properties. This hydrogel can also show stable mechanical properties after swelling under physiological conditions, which is a promising biomedical material. Ding et al. [110] developed a kind of self-healing hydrogel for wound dressing, composed of collagen, chitosan, and dibenzaldehyde-modified PEG_2000_ via dynamic imine bonds, and the collagen/chitosan hydrogels showed good thermal stability, injectability, and pH sensitivity, ideally promoting wound-healing performance and hemostatic ability (Figure 6a).

Aerogel is a three-dimensional porous nanomaterial in which the liquid component of hydrogel is replaced by gas during the drying process while the gel network is still maintained. The preparation of collagen aerogel is mainly divided into two parts. Firstly, the aqueous collagen gel is prepared using the acid swelling method, the water in the collagen gel is removed by drying, and the skeleton structure is retained to obtain collagen aerogel. In the preparation of the collagen aerogel, the drying method greatly affects the morphology of the aerogel, including the shape of the aerogel, the specific surface area, and the pore size distribution. In general, aerogel drying methods mainly include atmospheric drying, supercritical carbon dioxide drying, and freeze-drying. According to the unique properties of collagen material, freeze-drying is the most suitable drying method in the process of preparing collagen air coagulation. Yue et al. [111] proposed a promising strategy for preparing composite aerogels with pH-responsive properties based on collagen and TEMPO-oxidized cellulose nanofibers through the self-assembly behavior of collagen molecules for controlled drug release. Collagen fibrils and carboxylated cellulose nanofibers exhibited a high density and a physically cross-linked supramolecular double network structure, and cyclodextrins were introduced to optimize the structural stability and slow release capability of the composite aerogels. Zhang et al. [112] used cross-linked collagen fibers and MXene as raw materials to prepare a collagen/MXene composite aerogel with high sensitivity, rapid response, good thermal insulation performance, and stability by blending casting and freeze-drying methods (Figure 6b). Pot et al. [113] used a customized wedge system to regulate ice crystal growth through different freezing rates and suspension media, elucidated the mechanism of unidirectional ice crystal growth, and constructed a unidirectional collagen aerogel with adjustable pore size and morphology. Mekonnen et al. [114] synthesized collagen/polypyrrole hybrid aerogels with good biocompatibility and conductivity by in situ oxidation polymerization combined with freeze-drying methods, which have broad application prospects in biosensors, tissue engineering, electrostatic discharge protection, and electromagnetic interference shielding.

### 4.2. Modification of Collagen-Based Composite Materials

Pure collagen is directly used as a material with poor water solubility resistance, low mechanical properties, fast degradation in vivo, and easy erosion by bacteria. Therefore, in practical applications, collagen materials must be modified in certain ways to improve their mechanical strength of collagen materials, reduce the degradation rate, enhance water solubility resistance, etc., so as to broaden the application range of collagen materials [115]. At present, as shown in Table 1, collagen modification methods can be roughly divided into chemical modification and physical modification.

The chemical modification of collagen mainly includes cross-linking, grafting, copolymerization, and side chain modification. Among them, the cross-linking method, as the most widely used chemical modification method, refers to the cross-linking agent used to generate new chemical bonds between the end or side groups of the collagen polypeptide chain to achieve the purpose of cross-linking. The commonly used cross-linking agents are GA [107,116,117], carbonized diimide [119], and genipine [118], among which GA is the most widely used cross-linking agent. However, although GA has efficient cross-linking, its cytotoxicity is inevitable, which brings certain limitations to biomedical applications [125]. In recent years, researchers have paid more attention to cross-linking agents with low biotoxicity of carbonized diimide and genipine, which have been widely used in the study of chemical modification of collagen materials [126]. Perez-Puyana et al. [107] prepared collagen scaffolds using freeze-drying technology and cross-linked scaffold materials by GA, which reduced porosity and pore size and improved the mechanical properties of scaffolds. Luo et al. [127] cross-linked the collagen films prepared by electrospinning with 1-ethyl-(3-dimethylaminopropyl) carbanyl diimide and n-hydroxybutadiimide (EDC/NHS), GA, and genipine, effectively maintaining the shape of the wet films for two months and improving the mechanical properties under dry and wet conditions.

Although the chemical modification of collagen has a remarkable effect, it introduces other chemical agents into collagen materials, which leads to potential biological toxicity risks and has a certain impact on collagen’s original structure and properties. Therefore, physical modification without any chemical reagents has a broad application prospect in the field of collagen materials. The physical modification methods of collagen mainly include ultraviolet irradiation [124], severe dehydration (dehydrothermal cross-linking), and blending with other polymer materials [122,128,129]. Song et al. [124] made nanofilms with collagen modified by methacrylate anhydride through electrospinning technology and cross-linked it under ultraviolet irradiation, which greatly enhanced the water resistance and mechanical properties of the films. Andonegi et al. [106] blended chitosan with collagen with different molecular weights, prepared collagen/chitosan composite films by the solution pouring method, and evaluated the influence of chitosan incorporation with different molecular weights on the structure and properties of collagen films.

### 4.3. Application of Collagen-Based Composite Materials

At present, collagen-based composite materials are widely used in biomedicine, food engineering, beauty cosmetics, and other fields due to their excellent biological properties [130,131,132,133,134]. In addition, based on its unique fiber properties, collagen-based composite materials also have good applications in textile, papermaking, and other fields [135]. With the continuous development of future research, various excellent properties of collagen will be gradually discovered, and its application fields will be increasingly expanded.

#### 4.3.1. Biomedical Engineering

At present, natural biomedical polymer materials are grouped into two major categories: (1) natural polysaccharides, including cellulose, hyaluronan, chitosan, alginate, etc., and (2) natural proteins, including collagen, silk fibroin, fibrin, elastin, etc. [136]. Nevertheless, compared with natural polysaccharides, natural proteins have been widely used in biomedical engineering fields due to their biological origins, low immunogenicity, excellent biocompatibility, and good degradability [13,24,137,138]. The application of collagen-based composite materials in biomedicine engineering mainly includes hemostatic materials (such as sutures, wound dressings, etc.), tissue engineering materials (such as surgical implants, scaffolds, biofilms, etc.), and drug carrier materials (such as drug-carrying gels, granules, etc.) [24,139,140,141,142,143].

Collagen has active biological functions, which can actively participate in cell migration, differentiation, and proliferation; provide an ideal extracellular matrix environment for the growth of epidermal cells; play an important role in various stages of wound healing, including hemostasis, inflammation, hyperplasia, and remodeling; and contribute to the wound healing process [144]. The natural structure of collagen, especially the complete quaternary structure, is the basis of its coagulation effect. The free amino group in collagen (mainly the lysine side chain amino group) can induce platelet adsorption and aggregation, then activate coagulation factors to form thrombosis and prevent bleeding, and finally play a good role in hemostasis [145,146,147]. The main goal of tissue engineering is to regenerate or repair damaged, diseased, or eliminated organs and tissues using biocompatible and biodegradable scaffold materials [148]. Collagen has good biocompatibility, low immunogenicity, high biodegradability, and good mechanical, hemostatic, and cell binding properties, so it is an ideal scaffold material for tissue engineering applications [149]. In tissue engineering, collagen-based composite materials are mainly used for bone tissue repair and artificial tissue manufacture in vivo and in vitro [150,151,152]. Yang et al. prepared hydroxyapatit/mineralized collagen hydrogel composites by incorporating different amounts of dopamine-modified hydroxyapatit particles during the process of collagen self-assembly and simultaneous mineralization [153]. The results showed that the excellent adhesion properties of dopamine contributed to enhancing the bone repair potential of the composites, suggesting that mineralized collagen hydrogel composites containing dopamine-modified hydroxyapatit particles, which could mimic the structure and composition of bone, have a promising potential as bone tissue engineering scaffolds. Megha et al. developed a collagen scaffold coated with hydroxyapatite loaded with a peptide LL-37 [154]. The results confirmed that the hydroxyapatite coating on the outer and inner surfaces of the hydroxyapatite–collagen scaffold, and LL-37 loaded the hydroxyapatite–collagen scaffold and promoted the osteogenic differentiation of human osteosarcoma cells without showing significant cytotoxicity. In a drug-controlled release system, besides the drug itself, the drug carrier is also an important component. The drug’s carrier materials are mainly natural polymers and synthetic polymers. As a carrier material, collagen can regulate its degradability through cross-linking and use its different functional groups to customize the required materials [155,156]. The drug delivery system based on collagen can be prepared into films, tablets, sponges, particles, injections, and other shapes for different drug delivery sites and disease treatment [26,157]. Padekan et al. prepared a class of biocompatible composite hydrogel beads by using collagen and hydroxyapatite nanoparticles both extracted from fish wastes and chitosan for oral drug delivery [158]. The results showed that the mentioned hydrogel beads had a great potential delivery platform to achieve the controlled release of cefixime. Princy et al. prepared polycaprolactone/polyvinyl alcohol–collagen-based three-dimensional nanoscaffolds loaded with Cetirizine by the electrospinning technique [159]. The release trend of Cetirizine through these as-synthesized nanoscaffolds was analyzed by various kinetic models and exhibited a sustained release of Cetirizine for up to 96 h.

#### 4.3.2. Food Engineering

The unique properties of collagen give it incomparable advantages as a nutrient and functional material in food engineering. Collagen is rich in various amino acids, with the contents of proline and hydroxyproline being the highest among all kinds of proteins. Hydroxyllysine is not found in other proteins, so it is very rich in nutrition and can be used as a food in health food and food additives [160]. In addition, collagen, as a low-calorie, fat-free, and sugar-free animal protein, can accelerate the production of hemoglobin and red blood cells and maintain the normal function of blood vessels. It can be used as a health food to reduce weight, blood pressure, and blood lipid, which is conducive to preventing hypertension, arteriosclerosis, and other chronic diseases [161]. As a functional material, collagen is mainly used in food packaging materials and food coating materials in the food industry [102]. In recent years, all kinds of sausage products in meat products account for an increasing proportion, but natural casing products are seriously lacking. Collagen casings, dominated by collagen, are nutrient-rich and high-protein substances. In the heat treatment process, with the evaporation and dissolution of water and oil, collagen almost has the same shrinkage rate as meat. Artificial collagen casing also has the characteristics of good taste, high transparency, simple production process, and so on, which are conducive to mass production and good development prospects [162]. Collagen as a coating material used on food surfaces can avoid food oxidation; prevent powdery and granular sugary food from causing moisture absorption, clumping, and stiffness; and make the food surface glossy. In addition, collagen also has a preservative effect, which can be used as a stabilizer to form protective films on the surface of the food to prevent products from drying and shrinking, ensure the natural flavor and freshness of food, and improve the preservation of volatile food [163].

#### 4.3.3. Cosmetics

Collagen is the main component of human skin, accounting for about 72% of the dry weight of the skin. It is the main pillar to maintaining the mechanical properties of the skin so that the skin not only has protective properties but also has appropriate elasticity [132]. Collagen has excellent moisturizing properties and anti-free radical properties, which can protect the body cells from oxidation and have anti-aging effects [161]. However, with physiological age, the ability of fibroblasts to synthesize collagen begins to decline, and elastin becomes less elastic, slowly breaking down the regular network structure in the skin and eventually leading to wrinkles. Therefore, collagen-based materials used in beauty cosmetics can supplement a variety of amino acids needed by the skin, increase the activity of skin collagen, maintain the moisture of the stratum corneum and the stability of the fiber structure, improve the microenvironment of skin cells, promote the metabolism of skin tissue, and play a cosmetic role in moisturizing the skin, eliminating wrinkles, and delaying aging [133]. Collagen cosmetics are mainly creams and masks with high moisturizing properties, with special properties such as nutrition, repair, moisturizing, affinity, and compatibility [164]. When applied to the skin, collagen cosmetics cover it, reducing moisture loss and protecting it from corrosive elements. Collagen fillers are also used in cosmetic medicine. Subcutaneous injection of soluble collagen fillers can improve the quality and density of the skin and repair wrinkles and loose contouring. In addition, hydrolyzed collagen peptides are also widely used in some cosmetics. Collagen peptides are highly soluble in water, have moisturizing and antioxidant properties, and can penetrate deeper into the skin, making skin regeneration possible [160].

#### 4.3.4. Others

Controlled pyrolysis can transform collagen-rich biomass materials into collagen-derived porous carbon materials. The ordered biological structure and special natural precursor element composition (C, N, O, S, etc.) give the collagen-based porous carbon materials a unique nanostructure and heteroatomic doping, which give them a broad application prospect in electrochemical energy storage and conversion [165]. To date, collagen-based porous carbon materials are excellent carbon carriers and have been used in high-performance electrodes and electrocatalytic reactions in several electrical energy storage devices, such as lithium-ion batteries and sodium-ion batteries [166]. In addition, abundant surface atoms and a high specific surface area stratified porosity make collagen-based porous carbon materials have broad application prospects in photocatalysis, microwave adsorption, gas adsorption, dye adsorption, capacitive desalination, and solar steam power generation [165,167,168,169,170].

The application of collagen in the paper industry can be divided into fiber form and non-fiber form. Fiber form collagen is mainly used to compound with other plant fibers to improve paper performance and produce paper for special purposes in the printing industry. The carboxyl group, amino group, and hydroxyl group in collagen fibers can form hydrogen bonds and ionic bonds with cellulose in plant fibers to covalent bonds so that the binding force between fibers increases, thus improving the mechanical strength, air permeability, and water absorption of paper. The carboxyl group, amino group, and hydroxyl group in collagen fibers can form hydrogen bonds and ionic bonds with cellulose in plant fibers to covalent bonds so that the binding force between fibers increases, thus improving the mechanical strength, air permeability, and water absorption of paper. Non-fiber form collagen is rarely used and is usually used to develop adhesives, surfactants, flocculants, sizing agents, and other paper auxiliaries to improve paper properties.

The mechanical properties (especially the wet strength) and water solubility resistance of pure collagen fibers are poor, and the extraction cost is high, so it is not easy to promote in the textile field on a large scale. Blends with other polymeric materials such as polyvinyl alcohol, cellulose, polyethylene glycol, and hyaluronic acid are the most common applications of collagen in textiles. In addition, due to the high-temperature process commonly required in producing textile fibers, collagen in existing collagen-containing fabrics on the market has little biological activity. Therefore, maintaining collagen activity on fabric will be the focus and technical difficulty of future collagen research in the textile field.

## 5. Conclusions

To cope with a series of environmental problems brought on by global warming, the industrial structure of the traditional manufacturing industry is developing towards scale, intensification, and high performance through deep adjustments. As the most abundant protein in mammals, collagen has been closely related to human development. It is imperative to fully use animal husbandry’s abundant animal skin biological resources and develop new collagen products with high added value. In this review, we present the factors influencing the self-assembly process of collagen in vitro and some measurements to characterize the fibrillogenesis process in terms of light transmittance, morphologies, and other profiles and for the preparation, characterizations, and applications of collagen-based materials However, on the one hand, the existing collagen materials generally have shortcomings such as low mechanical properties, poor thermal stability, weak water solubility resistance, and fast enzyme degradation rate. On the other hand, it is difficult for the traditional collagen material preparation process to take into account the bottom-up hierarchical self-assembly behavior of collagen and the interaction between self-assemblies, so the mutual adaptation of collagen material structure and function cannot be realized in a real sense. Future perspectives encourage the fabrication of controllable collagen-based materials by modulating self-assembly conditions and the development of characterization methods that reveal other profiles during collagen fibrillogenesis. It is promising that such a compilation of recent strategies for controlling and characterizing collagen in vitro fibrillogenesis provides some insights into the self-assembly mechanisms and the design and fabrication of collagen-based materials for biomedical engineering, food engineering, cosmetics, and other applications.

## Figures and Tables

**Table 1:** Classification of modification methods for collagen.

	Classification	Possible Mechanism	Ref.
Chemical modification	GA	GA–protein cross-links are formed through reaction of ε-amine groups of lysine or hydroxylysine residues with the aldehyde group of GA.	[107,116,117]
	Genipin	(i) Fast nucleophilic attack of amine groups on lysine and arginine on the C3 atom of genipin, which leads to the formation of a heterocyclic compound of genipin connected with basic residues of proteins; (ii) Slow nucleophilic substitution of the ester groups in genipin which produces a secondary amide connection.	[118]
	EDC/NHS	The cross-linking reaction of collagen with the use of EDC/NHS induces the formation of a covalent bond between carboxylic acid groups from aspartic and glutamic acid.	[119]
	Dialdehyde starch	Dialdehyde starch aldehyde groups interact with the free amino group of collagen during the cross-linking reaction.	[120]
Physical modification	Dehydrothermal	Under vacuum conditions, water molecules are removed which causes the formation of intramolecular links (amide bonds) between collagen.	[121,122,123]
	UV light	This process involves the formation of free radicals on aromatic groups of tyrosine and phenylalanine, and radicals interact with each other and form chemical bonds form.	[124]

## Data Availability

Data sharing is not applicable.
